# The Intermittent Infectiousness of Scarlet Fever

**Published:** 1909-02-27

**Authors:** William Butler

**Affiliations:** Medical Officer of Health for Willesden


### SPECIAL ARTICLE.

### THE INTERMITTENT INFECTIOUSNESS OF SCARLET FEVER.

By WILLIAM BUTLER, M.B., C.M. (Glasgow), D.P.H.; Medical Officer of Health for Willesden.

(A Paper read before the Epidemiological Section of the Royal Society of Medicine.)

Scarlet fever loses nothing of its interest because of the purely inferential character of its hypothetical contagium. Doubtless, difficulty of interpretation is increased by reason of our ignorance concerning the *materies morbi*, but the phenomena which have eluded the promise of bacteriological research remain none the less a proper object of epidemiological interest. The nature of its infectiousness must, for the time being, be described in terms of its epidemical behaviour rather than be accounted for in the results of experimental investigation.

It is a feature of this behaviour—its intermittency—to which in particular I desire to call attention. Intermittent infectiousness is exhibited whenever a person who, between times, is innocuous becomes capable of infecting others with whom he may have been in contact. Perhaps the condition is best illustrated in the example of a common cold. Catarrhal affections of the naso-pharynx have at one time been ascribed to chill, at another to infection, and both accounts of the cause of "colds" are probably correct. Whether a "cold" be a specific zymotic disease or not, it is undeniably at times contracted by contact-infection, but the instances in which it is caught as a result of exposure to chill are too well authenticated, both by common and skilled observation, to admit of doubt as to this method of origin. However contracted, colds are, without doubt, infectious when set up. But colds contracted as a result of exposure to chill are examples of disease induced by a critical lowering of resistance in the presence of an infective agent to which, in the absence of such a crisis, the body is immune. The innocuous presence of the infective agent is the predisposing but essential condition of the irruption of infectivity, and a person who habitually lives in co-partnership with such infectious material may be said, with each recurrence of the exciting cause, to be intermittently infectious.

The flora of the naso-pharynx may be assumed normally to include organisms capable, in those states of body which follow upon chill, of becoming toxic. Having, for the nonce, acquired this property in respect of their habitual host, they preserve the same virulent character when planted out among contacts, with whom association hitherto had been followed by no such untoward consequences. By grouping kindred phenomena we may the better understand the identical process which they exemplify, and in the example of catarrhal fever just outlined we have a type to which, as I hope to show, scarlet fever conforms in many respects.

Erysipelas admittedly exhibits a selective incidence upon those previously attacked. A chill, a prick, a scratch, even a surgical wound made under aseptic precautions, may be followed in those who already have previously reacted to the erysipelatous streptococcus by another attack of the disease. When this happens such infected persons become possible foci of infection to others rendered susceptible by surgical, puerperal, or accidental injury. Yet another example is presented in a definitely specific infection—gonorrhœa. It may be years after the urethritis or other evidence of infectiousness has subsided that an indiscretion of the table, a severe chill, or other depressing circumstance lights afresh the old trouble, with an accompanying recurrence of the infectious state. Such cases are instances of intermittent infectiousness. Between the attacks there is extended a period of latency during which the tissues must be assumed to have harboured a quiescent infective material, innocuous to host or others, until injury lowered resistance and set free the restrained virulence of the lurking organism.

It may be urged that in the case of scarlet fever it is unnecessary to assume an intracorporeal habitat for the infective material, that infected fomites afford a sufficient reservoir on which to draw in explanation of almost all conceivable instances of apparent intermittent infectivity. It would be futile to attempt to prove the negative of this proposition. The view that infected fomites are a means by which scarlet fever is diffused is a theory which stands or falls accordingly as it offers an harmonious explanation of the facts. Without denying the occasional correctness of this explanation, I shall submit a view which it will be my endeavour to show is more generally consistent with the phenomena observed.

But first I should like to record a case which exemplifies the ineffectiveness of exposure to infected fomites of a person demonstrably susceptible to the disease. A parlourmaid, who for six months had been in daily attendance upon the nurses from the scarlet fever and diphtheria wards, left the Willesden Hospital, and two days afterwards was admitted to one of the Metropolitan Asylums Board's hospitals suffering from scarlet fever. The day on which she left Willesden a laundry-maid in the hospital was found to be suffering from scarlet fever, the rash having developed on that day. The previous day, while suffering from sore throat, she had been in intimate contact with the parlourmaid who had left, and there could be little doubt that this was the origin of the parlourmaid's attack. Six months' intimate personal contact with nurses fresh from the wards in their infected garments had failed to convey the disease to the parlourmaid, yet she succumbed immediately she came in contact with the actual disease. In fact, it has probably become necessary to expand the notion of the conditions under which personally conveyed infection may occur. The prolonged duration of personal infectiousness in those attacked by scarlet fever was for long obscured by the explanation that the infection clung to surfaces and fomites and retained its activity for indefinite and enduring periods. It may be that such is the case, but return cases of scarlet fever are not now explained as being due to the rehabilitation of some long-disused article of play or clothing which had escaped disinfection at the time of removal of the patient. The hypothesis that the infection is retained in and emanates from the persons of those who have recently recovered from the disease is now generally accepted. Whether the infectiousness of such persons is prolonged continuously in anomalous particular cases or is only intermittently persistent under circumstances for the most part undefinable are alternative though not necessarily antagonistic views. Without attempting a comprehensive statement, I believe the following to be true of the infectiousness of scarlet fever:—

1. Under varying but occasionally recognisable conditions persons recovered from scarlet fever are capable of conveying the infection to others after intervals frequently of prolonged duration, when apparently they have ceased to be infectious.

2. There are persons who have not suffered from the disease but appear to harbour the infection in their tissues in such manner that while there is no ground for considering them during this passive stage a danger to others they may yet, by a critical lowering of their resistance, become the source of their own infection.

3. There are others, again, of whom, while it would be incorrect to say they have suffered an attack of scarlet fever, have yet in some degree reacted to an invasion of the poison and are capable of communicating to those with whom they come in contact the disease to which as such they are themselves immune.

To take the last case first. The frequency with which sore throat in other members of a family precedes a fully developed attack in one of them leaves little room for doubt that such sore throat stands causally related to the attack in which the classical symptoms of the disease are unfolded.

In the following table is shown the frequency with which scarlet fever is associated with a history of sore throat in other inmates of houses invaded by the disease, together with control results of observations made in respect of diseases not naturally associated with sore throat.

Table showing Number of Houses in which persons had suffered from Sore Throat at, or within a month prior to, the time of inquiry.

| — | Scarlet Fever | | Other Diseases | |
|---|---|---|---|---|
| | No. of Infected Houses | No. of Houses where Sore Throat Occurred | No. of Houses where Inquiry was Made | No. of Houses where Sore Throat Occurred |
| Totals ... | 1,266 | 433<br>38* | 1,644 | 47 |
| Net ... | 1,266 | 395 | 1,644 | 47 |
| Percentage.. | | 31.2 | | 2.8 |

* In these thirty-eight instances the sore throat complained of at the time of inquiry subsequently proved to be scarlet fever.

The disparity in frequency in the two series leaves little room for doubt that the prevalence of sore throat in scarlet fever-invaded houses is due in some way to the scarlet fever outbreak itself. Such sore throats are probably identical with those from which nurses and attendants in scarlet fever wards have frequently been observed to suffer, and are to be looked upon as abortive attacks of the disease. This view is supported by consideration of the difference in age-incidence of scarlet fever and of the related sore throat. Although about 85 per cent. of the cases of scarlet fever occur in children aged under 15, the related sore throat occurs under 15 years of age in about 50 per cent. only of the total number affected. The greater resistance in those over 15 years of age exposed to attack presumably results in the initial symptoms alone developing, the attack being aborted at this stage. Diphtheria presents a demonstrable analogue to this condition. A transient sore throat in persons exposed to infection is followed by none of the classical symptoms of the disease, but bacteriological examination reveals the specific character of the faucial inflammation. During influenza epidemics many persons not otherwise ill suffer from a passing lumbago or other muscular pains, which must be regarded as definite reactions to the influenzal infection to which they have been subjected.

In epidemics of other diseases, such as typhoid fever or smallpox, mild cases of illness presenting one or other feature of the prevailing disease are to be found which, while not recognisable as instances of the disease in question, are yet in all probability capable of communicating it to others in its clinical completeness. Apart from the statistical evidence just given it is frequently difficult to account for the origin of certain attacks of scarlet fever, except upon the supposition that sufferers from sore throat are sometimes capable of conveying the disease to others when all traces of their indisposition have disappeared. The following is an instance—one among many I have noted—which serves to illustrate what I believe to be a far from uncommon occurrence:—

Roger N., removed to isolation hospital September 26, 1908, suffering from scarlet fever; onset September 25, rash September 26. Shortly before the onset of Roger's illness his brother Albert suffered from sickness and malaise, with redness of fauces, and was considered by his medical attendant to be possibly sickening with scarlet fever. However, as the symptoms passed off without the appearance of any rash, the diagnosis of scarlet fever was negatived. A week later (October 3, 1908) Albert visited his cousin, Ernest A., at a house some distance away, and four days later the cousin failed with scarlet fever and was removed to hospital on the following day. No contact of Ernest A. with Roger N. had occurred subsequent to the onset of Roger's attack of scarlet fever, and Ernest A.'s attack could only be accounted for on the supposition that Albert had acted as a carrier case, not having himself suffered from a recognisable attack of the disease. It is to be noted that Albert did not desquamate.

Even a more striking instance of the infectivity of a transient sore throat unaccompanied by other symptoms is furnished in the following group of cases. Towards the end of September of last year a sharp outbreak occurred in a public elementary school.

A number of children suffering from suspicious throats were excluded from school. Among these was a child, Kitty L. During her period of quarantine Kitty L. showed no other symptoms than the sore throat referred to, and she was readmitted to school fourteen days after the date of her exclusion, apparently in good health. Eight days after her readmission, however—on October 12—her brother was notified as suffering from what proved to be a fatal attack of scarlet fever, and on the same day a boy in the same class as Kitty L., George H., was also notified as suffering from the same disease. Both George H. and Kitty L.'s brother had an onset occurring on the same day, namely, October 9. There had been no other cases in the school within three weeks of George H.'s attack.

If the evidence be accepted that persons indiscernibly affected with scarlet fever are yet capable of communicating the disease to others, it will be seen how much more difficult and complicated is the problem of tracing the origin of attacks to infected fomites and surfaces. But more important than this widening of the epidemiological problem is the fact itself that persons presenting no evidence of suffering or of having ever suffered from scarlet fever may, notwithstanding, be harbouring the infectious material of the disease, and be the means of infecting others with whom they come in contact. The recognition that apparently normal persons may in their own bodies be carriers of scarlatinal infection very probably accounts for the occurrence of many cases of surgical scarlatina and diphtheritic attacks. It has frequently been noted, and my own observations confirm the fact, that the operation for the removal of adenoids and enlarged tonsils is apt to be followed by an attack of diphtheria or scarlet fever. The interval between the operation and the onset of symptoms so commonly corresponds with the normal incubative period of these diseases that it is difficult to believe that the patient has become infected subsequent to the performance of the operation, and what one is forced to think has happened is that the surgical interference has lowered resistance and auto-infection has been the consequence.

Post-operative scarlet fever is probably strictly analogous to post-operative diphtheria, and each condition presents the phenomenon of an aroused activity of infection due to an induced lowering of resistance. In such instances we have, perhaps, the simplest examples of an intermittent infectiousness, the intermittency depending upon some crisis in the normal resistance to infection. Modifications in the degree of susceptibility to specific contagia are responsible for quite as profound changes in the epidemiological history of diseases as are the alterations in virulence of the *materies morbi*, and the familiar example of small-pox stayed in its historic natural epidemicity by the induced immunity of vaccination is a striking instance of this significant fact.

In scarlet fever many of the epidemical features of the disease demand for their explanation less the finding of an actual preceding case than the recognition of variation in the degree of immunity in presence of a tissue infection which has long antedated the actual onset of the disease. It has frequently been observed that children admitted to the scarlet fever wards of a hospital, erroneously supposed to be suffering from the disease, have failed to contract it during possibly weeks of most definite exposure, and then have succumbed to the infection upon their discharge and return to the environment of home. It is also probably more than a coincidence that a considerable number of children discharged from hospital after recovery from either scarlet fever or diphtheria should have to be readmitted a few days afterwards from the alternate disease. In all such cases it is probable that the change from the hygienic conditions of the hospital to the less healthy circumstances of the home lowers resistance, and in children capable of auto-infection this variation in degree of immunity determines the attack. It is, on the other hand, no uncommon occurrence to find children attending school desquamating from scarlet fever, who have been in regular attendance during the whole course of their illness and yet have failed during what might be supposed to have been the most active infectious stage to communicate the disease to any of the inmates of the school, although later on they have shown themselves capable of spreading the disease. We are probably in such instances presented with exacerbations of infectivity in the primary case or cases, such exacerbations being disproportionate to the variations in resistance of those exposed. The following series of cases is selected, not merely as illustrating the operation of both these conditions, but as combining many other features in the epidemicity of scarlet fever:—

Florrie C. was taken ill with sore throat on November 5, 1907. She remained at home until November 11, when she returned to school and continued attendance until November 19, when she was reported as desquamating, and upon being examined was diagnosed as suffering from scarlet fever. No cases of scarlet fever were known to have occurred among the scholars attending the same public elementary school as Florrie C., and no cases of scarlet fever were directly traceable to contact with her prior to her admission to hospital on November 21. On the same date, however, as the commencement of Florrie C.'s illness, namely, November 5, 1907, a boy living next door, Albert E., also suffered from sore throat. He did not desquamate or present other symptoms of scarlet fever, but a month after Florrie C. had been removed to hospital, namely, on December 23, a sister of Albert E., Hilda E., failed with scarlet fever. Her attack could only be accounted for on the supposition that the brother Albert had acted as a carrier case, careful inquiry failing to discover other possible source of infection. During her stay in hospital Florrie C. was found not only to be suffering from scarlet fever, but to be harbouring Klebs-Lœffler bacilli. She was discharged from hospital on February 23, 1908, and about a fortnight later, on March 8, her brother, Arthur C., failed with scarlet fever. Arthur's illness was looked upon as being in the nature of a return case and the first to derive from Florrie C.'s attack, notwithstanding her attendance at school during the early part of her illness. On March 12 (four days after the onset of Arthur's illness) Harriet C., a sister of Florrie's, also failed of scarlet fever, and on the same day John K., a lodger in the same house, failed with diphtheria. John K. was discharged from hospital on April 6, Harriet C. on April 25, and Arthur C. on May 2. On May 6 John K. was again admitted to hospital, having failed with scarlet fever the previous day. On May 9 Nellie J., living in the same street, failed with scarlet fever, and it was ascertained she had been in intimate contact with Harriet C. On May 11 Mrs. E., the mother of Albert and Hilda E., already referred to, failed with scarlet fever. She had been looking after Harriet C. since the latter's return from hospital. On May 12 Hilda E., whose previous attack on December 23 has been mentioned, again failed with scarlet fever, and on May 19 Albert E., the supposed carrier in Hilda's attack, also failed of the disease. Nellie J., whose onset on May 9 was believed to be due to contact with Harriet C., was discharged from hospital on July 4, and on July 21 her cousin, Harry J., living in the same house, failed with scarlet fever.

I do not propose to comment in detail upon these cases. Nothing in the course of the inquiries was ascertained which could modify the natural inference as to the origin of the respective cases which the bare record suggests. I will, however, refer to another series of cases which illustrates in lesser degree the intermittent character of scarlatinal infectiousness:

In January 1908 two cases of scarlet fever occurred at a house in Harrow, and the medical officer of health wrote me that two children, Gladys and Florence Y., living in Willesden, had attended a party on January 6, one of them being stated recently to have suffered from sore throat. Suspicion attached to the party as the probable origin of the outbreak. Upon visiting the address in Willesden where Florence and Gladys Y. lived, I found that both children were at school—Gladys at one school, Florence at another. When seen, both children were found to be desquamating, and inquiry elicited an onset of the disease on December 26 and 27 respectively. They attended large public elementary schools up to January 16. No cases of scarlet fever occurred at the school attended by Gladys, but one case occurred at the school attended by Florence the day after she ceased attending. In addition to this case, however, it was ascertained that three young adults, aged respectively 18, 27, and 24, who visited at the house of Florence and Gladys, and were frequently visited by them, were each attacked with scarlet fever, the onset of the first case being January 13 and of the other two January 25 and 27.

I will quote only one other instance illustrating the infectivity of persons not discernibly suffering from scarlet fever:—

Lily J. was excluded from attendance during a school outbreak of scarlet fever on account of suspicious illness. There was malaise on September 19, and vomiting, sore throat, and fever on September 20. No rash was observed and no desquamation followed, although she was kept under observation for over a month. Four days after the onset of Lily's illness her brother Arthur was taken ill with headache, lassitude, and feverishness. No rash was observed, but three weeks afterwards he commenced a typical scarlatinal desquamation. His initial symptoms were much less characteristic and severe than were those of his sister, yet he alone presented the diagnostic features of the disease.

It is a very common experience to find that after cases have been removed to hospital the disease breaks out afresh in the household long after the recognised incubative period has elapsed. Many such cases are doubtless due to the retention at home of overlooked or aborted cases; but it so frequently happens, where the most careful search fails to discover such possible infecting cases, that one is impelled to fall back upon the possibility of auto-infection of the patient at a late period due to some critical fall in his resistance or exacerbation of the latent infection he is harbouring. The following cases illustrate this point:—

Ethel W., removed to isolation hospital suffering from scarlet fever, September 21, 1908; rash, September 20, 1908. Arthur W., in contact with Ethel up to the date of her removal, remained well until October 11, 1908, when he developed a "cold," and on October 16 he failed with scarlet fever, the rash appearing the following day. When he was removed to hospital on October 18 his brother John also failed with scarlet fever, the rash appearing on October 19. No other persons in the house had suffered from sore throat or other suspicious illness before or after the removal of Ethel to hospital, and it is probable that the superadded infection of a common cold so reduced Arthur's resistance to the infection, which presumably he had harboured since Ethel's removal, that he contracted the disease by auto-infection and then communicated it to his brother.

A history of superadded infection, such as common cold or influenza, occurring in cases of this character a few days prior to the onset of scarlet fever is so frequent as to suggest the probability of its being an element in the causation. Such added infection conceivably may act by lowering the specific resistance to an infection already present in the tissues, or by symbiotic process raising such latent infection to a pitch of critical virulence. Whichever be the *modus operandi* of infection delayed beyond the commonly ascribed incubative period, there can be no question as to the frequency of its occurrence.

In the following table is shown an analysis of 459 consecutive cases occurring in houses previously infected. The cases are respectively distributed according to the intervals which elapsed between their dates of onset and the dates of removal to hospital of the last preceding case in the house. The table is thus constructed from records of cases where the latest date of possible exposure is fixed by the isolation of the patient.

Table showing Intervals between Removals and Occurrence of Secondary Cases, and Number of Cases occurring at Respective Intervals.

| Interval | No. of Cases |
|---|---|
| 1 to 4 days ... ... ... | 210 |
| 4 ,, 7 ,, ... ... ... | 70 |
| 7 ,, 10 ,, ... ... ... | 31 |
| 10 ,, 14 ,, ... ... ... | 28 |
| 14 ,, 28 ,, ... ... ... | 39 |
| 28 days to 2 months ... ... | 53 |
| 2 to 3 months ... ... ... | 28 |

The figures permit of many inferences, but it can hardly be questioned that they mean the failure to remove, with the isolation of the patient and disinfection of the premises, all sources of infection in houses invaded by scarlet fever. The prolonged duration of this residual infection and the gradual diminution of its activity with the lapse of time are strongly suggestive that this infection is stored in the tissues of the contacts and recrudesces with a lessening frequency as its origin becomes remote. Whether such contacts are dangerous to others or only to themselves, during the prolonged quiescence of the infection, would seem to be answered mainly by the fact that when the patient fails after such a period of prolonged quiescence he usually infects others who have with impunity been in contact with him during the latent phase of the infection. For a month at least after the removal of infected cases from a household the centrifugal diffusion of the infection can be traced. Beyond that period the influence of the primary case or cases in the household is obscured in the period to which the figures relate by the return of the primary cases after a period of isolation. With their return is reintroduced a possible centre of further diffusion, and it is probable that the recurrence of scarlet fever in houses after this period is due largely to such reinforcement.

(*To be Concluded.*)

